# Heterogeneity of Multifunctional IL-17A Producing *S*. Typhi-Specific CD8+ T Cells in Volunteers following Ty21a Typhoid Immunization

**DOI:** 10.1371/journal.pone.0038408

**Published:** 2012-06-05

**Authors:** Monica A. McArthur, Marcelo B. Sztein

**Affiliations:** 1 Center for Vaccine Development, University of Maryland School of Medicine, Baltimore, Maryland, United States of America; 2 Department of Pediatrics, University of Maryland School of Medicine, Baltimore, Maryland, United States of America; Federal University of São Paulo, Brazil

## Abstract

*Salmonella enterica* serovar Typhi (*S*. Typhi), the causative agent of typhoid fever, continues to cause significant morbidity and mortality world-wide. CD8+ T cells are an important component of the cell mediated immune (CMI) response against *S*. Typhi. Recently, interleukin (IL)-17A has been shown to contribute to mucosal immunity and protection against intracellular pathogens. To investigate multifunctional IL-17A responses against *S*. Typhi antigens in T memory subsets, we developed multiparametric flow cytometry methods to detect up to 6 cytokines/chemokines (IL-10, IL-17A, IL-2, interferon-γ (IFN-γ), tumor necrosis factor-α (TNF-α) and macrophage inflammatory protein-1β (MIP-1β)) simultaneously. Five volunteers were immunized with a 4 dose regimen of live-attenuated *S*. Typhi vaccine (Ty21a), peripheral blood mononuclear cells (PBMC) were isolated before and at 11 time points after immunization, and CMI responses were evaluated. Of the 5 immunized volunteers studied, 3 produced detectable CD8+ T cell responses following stimulation with *S*. Typhi-infected autologous B lymphoblastoid cell lines (B-LCL). Additionally, 2 volunteers had detectable levels of intracellular cytokines in response to stimulation with *S*. Typhi-infected HLA-E restricted cells. Although the kinetics of the responses differed among volunteers, all of the responses were bi- or tri-phasic and included multifunctional CD8+ T cells. Virtually all of the IL-17A detected was derived from multifunctional CD8+ T cells. The presence of these multifunctional IL-17A+ CD8+ T cells was confirmed using an unsupervised analysis program, flow cytometry clustering without K (FLOCK). This is the first report of IL-17A production in response to *S*. Typhi in humans, indicating the presence of a Tc17 response which may be important in protection. The presence of IL-17A in multifunctional cells co-producing Tc1 cytokines (IL-2, IFN-γ and TNF-α) may also indicate that the distinction between Tc17 and Tc1 responses in humans is not as clearly delineated as suggested by *in vitro* experiments and animal models.

## Introduction

*Salmonella enterica* serovar Typhi (*S*. Typhi), a human restricted pathogen, is the causative agent of typhoid fever. *S*. Typhi is a Gram-negative, facultative intracellular pathogen that poses a significant threat to public health, particularly in the developing world [1], [2]. Typhoid fever is responsible for an estimated 21 million illnesses and 200,000 deaths annually and increasing antibiotic resistance among *S*. Typhi isolates has further exacerbated the problem [1], [2], [3], [4], [5], [6], [7], [8]. T cells, and particularly CD8+ T cells are thought to play an important role in the immune response against *S*. Typhi by producing interferon-γ (IFN-γ) and other T helper (Th) 1/T cytotoxic (Tc) 1 cytokines [9], [10], [11], [12], [13], [14], [15], [16], [17], as well as killing *S*. Typhi infected cells [10], [18]. Additionally, multifunctional CD8+ T cells have been identified in volunteers immunized with live-attenuated typhoid vaccine Ty21a [13]. Of note, the latter study has shown multiphasic cytokine production by CD8+ T cells in response to human leukocyte antigen E (HLA-E)-restricted antigenic stimulation [13].

Interleukin-17A is a pro-inflammatory cytokine that is involved in protection against extracellular and intracellular bacterial pathogens, as well as fungi and viruses [19], [20], [21], [22], [23]. IL-17A and associated Th17 cytokines promotes neutrophil recruitment via induction of chemokines and granulopoietic factors [19], [23]. Additionally, IL-17 has been shown to regulate Th1 responses by altering IL-12p70 production by dendritic cells [24]. In addition to Th17, the presence of CD8+ Tc17 cells that produce IL-17A have recently been described [25], [26], [27], [28].

Therefore, to evaluate the hypothesis that IL-17A may play a role in protection from *S.* Typhi infection, we investigated the presence of IL-17A producing CD8+ T cells in peripheral blood mononuclear cells (PBMC) obtained from Ty21a recipients following *ex vivo* stimulation with *S*. Typhi-infected autologous B-lymphoblastoid cell lines (B-LCL) or an *S*. Typhi-infected HLA-E restricted cell line. Furthermore, given the prominent role that multifunctional T cells have been shown to have in the host defense from other human infections [29], [30], [31], we investigated the concomitant production of IL-17A with 5 other cytokines/chemokines (IL-10, IL-2, IFN-γ, tumor necrosis factor-α (TNF-α), and macrophage inflammatory protein-1β (MIP-1β) as a first step to investigate the potential role of multifunctional T cells in protection from *S.* Typhi infection.

## Materials and Methods

### Volunteers and Isolation of Peripheral Blood Mononuclear Cells (PBMC)

PBMC collected from 6 healthy adult volunteers, recruited from the Baltimore-Washington area and University of Maryland, Baltimore campus, were used in this study. Written informed consent was obtained and all procedures were approved by the University of Maryland, Baltimore IRB. PBMC were isolated immediately after blood draws by density gradient centrifugation and cryopreserved in liquid nitrogen following standard techniques [18], [32].

### Target/stimulator Cells

Epstein Barr virus (EBV)-transformed B-LCL were generated from PBMC obtained from Ty21a vaccinees and control volunteers as previously described [18]. Briefly, B-LCL were established using supernatants from the B95.8 cell line (ATCC CRL1612; American Type Culture Collection) as the source of EBV. PBMC from each volunteer were incubated with EBV containing supernatant and cyclosporine (0.5 µg/mL; Sigma, Saint Louis, MO) at 37°C with 5% CO_2_ for 2–3 weeks. B-LCL were maintained in culture or cryopreserved until use.

An HLA class I-defective B cell line transfected with HLA-E fused to the HLA-A2 leader peptide (therefore expressing the HLA-E*0101 allele, but not HLA-A, -B, -C on the cell surface), 721.221.AEH (AEH cells), were provided by Dr. D. Geraghty [9], [13], [33]. AEH cells were maintained in RPMI 1640 media (Gibco, Carlsbad, CA) supplemented with 100 U/mL penicillin (Sigma), 100 µg/mL streptomycin (Sigma), 50 µg/mL gentamicin (Gibco), 2 mM L-glutamine (Gibco), 10 mM HEPES buffer (Gibco), 10% fetal bovine serum (Gemini Bioproducts, West Sacramento, CA), and 100 µg/mL hygromycin (Sigma).

### Infection of Target/stimulator Cells

Target cells were infected by incubation with wild-type *S*. Typhi strain ISP1820 in RPMI without antibiotics for 3 hours at 37°C with 5% CO_2_ as previously described [18]. On the day following infection the cells were gamma irradiated (6000 rad). To confirm that targets were infected with *S.* Typhi, cells were stained with anti-*Salmonella* common structural Ag (CSA-1)-FITC (Kierkegaard & Perry, Gaithersburg, MD) and analyzed by flow cytometry using an LSR-II instrument (BD). The percentage of cells infected with *S*. Typhi was recorded for each experiment. Infected targets were only used if infection was >30% of viable cells.

### Ex vivo Stimulation

PBMC were thawed and rested overnight at 37^o^C, 5% CO_2_ in complete media (RPMI 1640 media (Gibco) supplemented with 100 U/mL penicillin (Sigma), 100 µg/mL streptomycin (Sigma), 50 µg/mL gentamicin (Gibco), 2 mM L-glutamine (Gibco), 2.5 mM sodium pyruvate (Gibco), 10 mM HEPES buffer (Gibco), and 10% fetal bovine serum (Gemini Bioproducts,)) at a concentration of 1×10^6^ cells/mL in 6 or 12 well plates. Cells were then resuspended in complete media and stimulated with *S*. Typhi-infected autologous B-LCL or *S*. Typhi-infected HLA-E restricted AEH cells. Media alone and uninfected autologous B-LCL or HLA-E restricted AEH cells were used as negative controls. Staphylococcal enterotoxin B (SEB) (10 µg/mL; Sigma) was used as a positive control. Cells were incubated at 37°C in 5% CO_2_. After 2 hours, Golgi Stop (containing monensin) and Golgi Plug (containing brefeldin A) from BD were added at concentrations of 0.5 µl/mL and cultures continued overnight at 37°C in 5% CO_2_.

### Surface and Intracellular Staining for Flow Cytometric Analyses

Following stimulation as described above, cells were plated in 96-well V-bottom plates for staining. Cells were washed once with staining buffer (phosphate buffered saline with 0.5% BSA and 0.1% sodium azide) and stained for live/dead discrimination using Invitrogen LIVE/DEAD fixable violet dead cell stain kit (Invitrogen, Carlsbad, CA). Fc receptor blocking was performed with human immunoglobulin (3 µg/mL; Sigma) followed by surface staining, performed as previously described [13]. Briefly, cells were stained with CD14-Pacific Blue (TuK4, Invitrogen) and CD19-Pacific Blue (SJ25-C1, Invitrogen), CD3-biotin (UCHT1, Beckman Coulter, Danvers, MA), CD4-Qdot 655 (S3.5, Invitrogen), CD8-Qdot 705 (3B8, Invitrogen), CD45RA-Qdot 605 (MEM56, Invitrogen), and CD62L-PE-Cy5 (Dreg 56, BD, Franklin Lakes, NJ) at 4°C for 30 minutes. Staining with streptavidin-Pacific Orange (Invitrogen) was performed for panels that included biotin-conjugated monoclonal antibodies for 30 minutes at 4°C. The cells were then fixed and permeabilized using IC fixation and permeabilization buffers from eBiosciences according to manufacturer’s recommendations. Intracellular staining with IL-17A-PerCP-Cy5.5 (eBio64DEC17, eBioscience), IL-2-PE-Cy7 (MQ1-17H12, BD), IFN-γ-Alexa 647 or APC-Alexa 750 (B27, BD or Invitrogen), TNF-α-Alexa 700 (MAb11, BD), IL-10-FITC (127107, R&D, Minneapolis, MN), MIP-1β-APC (24006, R&D), and CD69-PE (FN50, eBioscience) was performed at 4°C overnight. After staining, cells were fixed in 1% paraformaldehyde and stored at 4°C until analyzed. Cells stained with the 13-color panels described above were analyzed by flow cytometry using a customized LSRII flow cytometer (BD). Flow cytometry data were analyzed using WinList version 7 (Verity Software House, Topsham, ME) and FLOCK (ImmPort, NIH web site www.immport.org) software packages. Graphs were generated using GraphPad Prism version 5.03 (Graphpad Software, San Diego, CA).

### Statistical Analyses

All tests were performed using GraphPad Prism version 5.03 (Graphpad Software). Comparisons between groups were performed using Mann-Whitney and Kruskal-Wallis tests. In flow cytometry experiments a response was considered significant if the differential in the number of positive events between experimental (*S.* Typhi-infected) and negative control (non-infected) cultures was significantly increased by Chi-square tests. P values <0.05 were considered significant.

## Results

### Kinetics of S. Typhi-specific CD8+ T Cells in Response to Stimulation with S. Typhi-infected Autologous Cells

**Figure 1:**
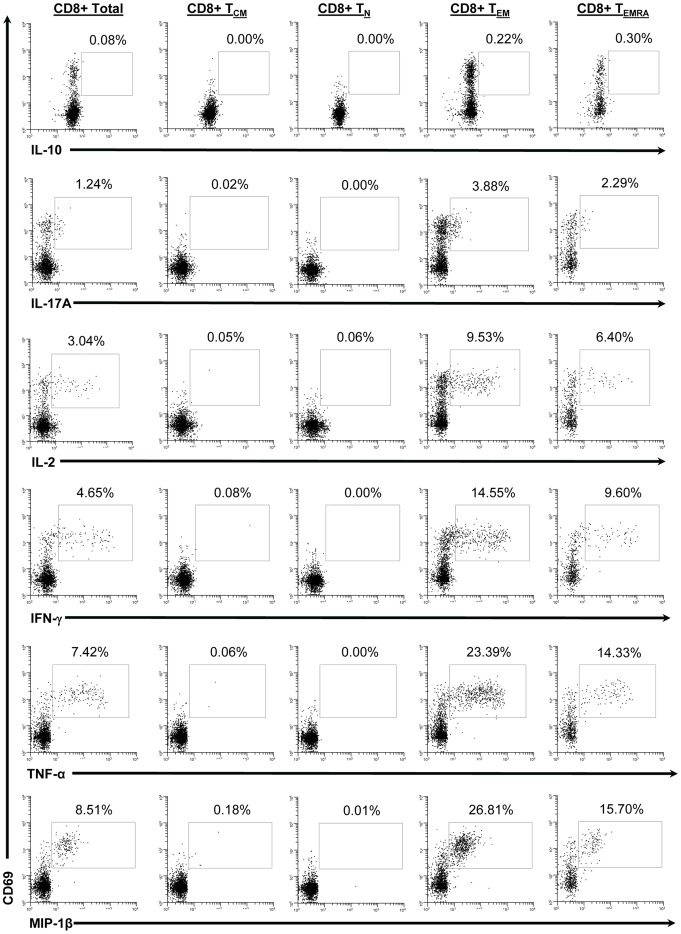
Detection of intracellular cytokines/chemokines produced by CD8+ T cell memory subsets in response to stimulation with *S.* Typhi-infected autologous B-LCL. Histograms from a representative volunteer (53 s at day 7 post infection) showing the production of 6 cytokines/chemokines by memory T cell subsets following stimulation with *S*. Typhi-infected autologous B-LCL. CD69 (a marker of recent activation) is displayed on the y-axis and each of the 6 cytokines/chemokines measured are displayed on the x-axes. Column 1 represents total CD8+ T cells, column 2 represents T central memory (T_CM_; CD62L+ CD45RA-), column 3 represents T naïve (T_N_; CD62L+ CD45RA+), column 4 represents T effector memory (T_EM_; CD62L- CD45RA-), and column 5 represents T effector memory CD45RA+ (T_EMRA_; CD62L- CD45RA+). The percentage of positive cells is shown for the indicated region in each cytogram.

Previous studies have indicated that CD8+ T cells are a major component of the CMI response to *S*. Typhi [10], [14], [15], [18]. Therefore we set out to determine the kinetics of CD8+ T cell responses to *in vitro* stimulation with autologous *S.* Typhi-infected cells following Ty21a immunization. Five healthy adult volunteers received 4 doses of the licensed oral, live-attenuated *S*. Typhi vaccine Ty21a on days 0, 2, 4, and 7. One volunteer did not receive Ty21a (negative control). PBMC were isolated before immunization and at 11 time-points up to 1 year after immunization (days 2, 4, 7, 10, 14, 28, 42, 56, 120, 180, and 360). Following isolation, PBMC were stimulated with *S*. Typhi-infected autologous B-LCL. Uninfected B-LCL and SEB were used as negative and positive controls respectively. PBMC were stained with monoclonal antibodies against CD3, CD4, CD8, CD14, CD19, CD45RA, and CD62L followed by intracellular staining for CD69, IL-10, IL-17A, IL-2, IFN-γ, TNF-α, and MIP-1β. Due to unavailability of the antibody, MIP-1β staining was not performed in 2 of the volunteers (48 s and 50 s). The samples were then analyzed by multichromatic flow cytometry. The gating strategy is shown in Figure S1. CD3+ CD8+ T cells were categorized by their expression of CD62L and CD45RA into naïve T cells (T_N_; CD62L+ CD45RA+), T central memory cells (T_CM_; CD62L+ CD45RA-), T effector memory (T_EM_; CD62L- CD45RA-), and T effector memory CD45RA+ (T_EMRA_; CD62L- CD45RA+) as previously described (Figure S1) [34]. We observed the intracellular production of multiple cytokines to be predominantly by the T_EM_ subset with a smaller contribution by the T_EMRA_ subset (Figure 1).

**Figure 2:**
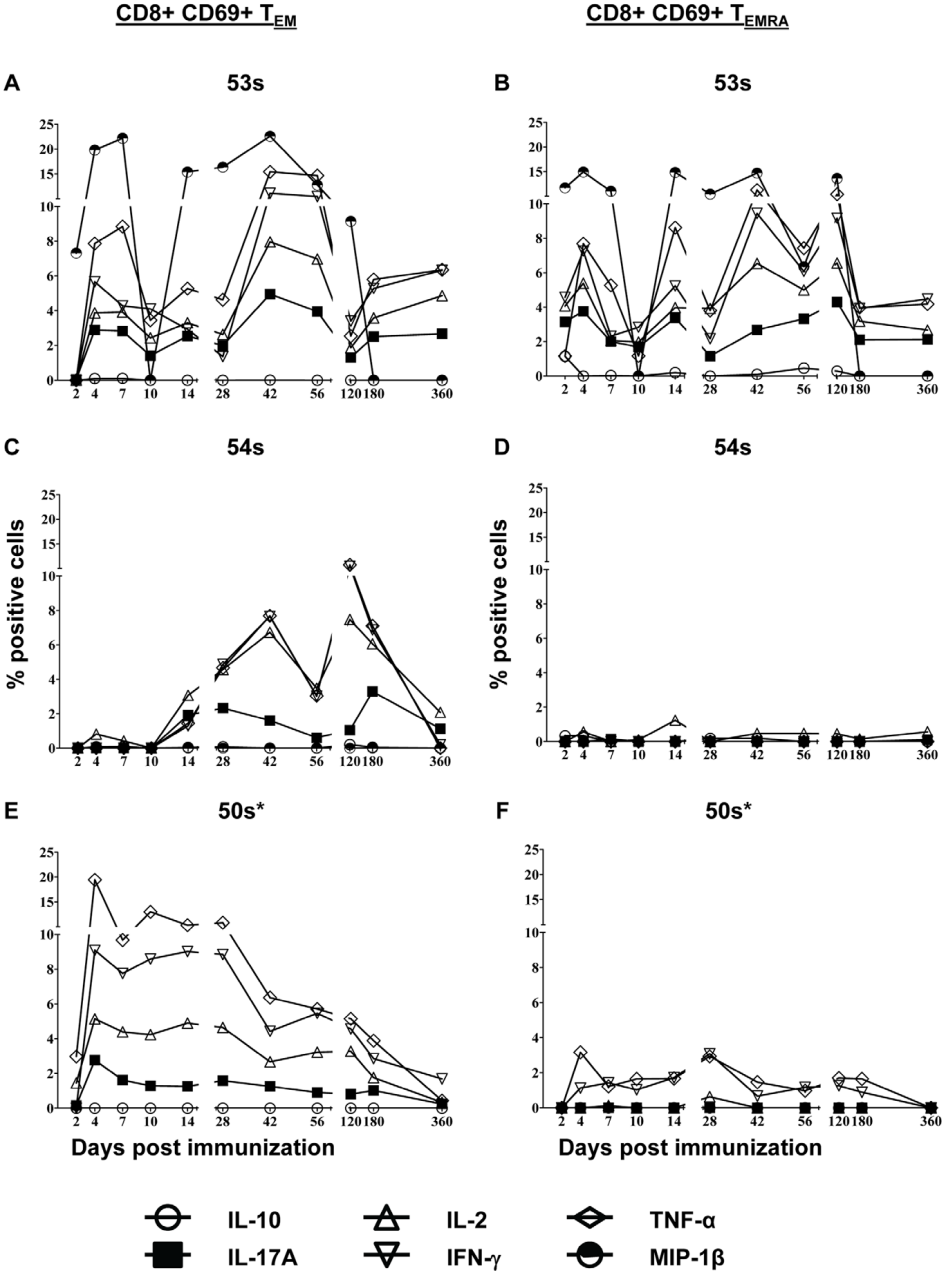
Kinetics of intracellular cytokine/chemokine production following stimulation of PBMC with *S*. Typhi-infected autologous B-LCL. Intracellular cytokine/chemokine production is shown as the percentage of positive cells. Responses are expressed as net values with day 0 subtracted to normalize for differences in base-line responses to *S*. Typhi in different volunteers. A) CD8+ T_EM_ cells for volunteer 53 s B) CD8+ T_EMRA_ cells for volunteer 53 s C) CD8+ T_EM_ cells for volunteer 54 s D) CD8+ T_EMRA_ cells for volunteer 54 s E) CD8+ T_EM_ cells for volunteer 50 s F) CD8+ T_EMRA_ cells for volunteer 50 s. Increases of >0.5% cytokine positive cells over uninfected targets were found to be consistently statistically significant (P<0.01) by chi-square analyses. * MIP-1b not measured.

Low levels of cytokine production were detected in PBMC stimulated with uninfected autologous B-LCL and this background was subtracted to calculate the *S*. Typhi-specific response. There was some variable baseline responsiveness on day 0 in most volunteers, likely due to cross-reactivity with other enteric Gram negative rods. Therefore, the net value (day x post immunization – day 0) was used to normalize the data. We found that 3 of the 5 immunized volunteers (60%) showed detectable intracellular cytokine responses above baseline responsiveness at day 0 (Figure 2 and Figure S2). This is consistent with our previous data using PBMC from subjects immunized with Ty21a and, interestingly, with most field studies which report a vaccine efficacy of 65–70% [10], [13], [35]. Intracellular IL-10 was not detected in any of the volunteers at any time-point. MIP-1β was measured in only 2 of the 3 responders (53 s and 54 s). There was significant production of MIP-1β by both T_EM_ and T_EMRA_ from volunteer 53 s (Figure 2 A & B); however, volunteer 54 s produced no detectable MIP-1β (Figure 2 C & D). CD8+ T_EM_ from all 3 volunteers (53 s, 54 s, and 50 s) produced high levels of TNF-α and IFN-γ (5–15% of circulating T_EM_ at the peak times), followed by IL-2 (4–8% of circulating T_EM_ at the peak times), and then IL-17A (2–5% of circulating T_EM_ at the peak times) (Figure 2). Intracellular IL-17A production was found in all 3 responders with kinetics that followed those of the other cytokines/chemokines measured (except MIP-1β) (Figure 2). The kinetics of the responses varied considerably among volunteers; however, all 3 volunteers showed bi- or tri-phasic responses. Early peaks were observed in volunteers 53 s and 50 s at days 4 to 7 post immunization (Figure 2 A, B, E, & F). In volunteer 53 s, additional peaks were observed at days 42 and 180–360 post immunization (Figure 2 A). The kinetics of MIP-1β differed from the kinetics of the other 5 cytokines with only 2 peaks at day 4 to 7 and days 28 to 56 (Figure 2 A). Volunteer 54 s had a bi-phasic response without an early peak. Peaks occurred at days 42 and 120 post immunization (Figure 2 C & D). In addition to the early peak at day 4 post immunization, volunteer 50 s demonstrated a peak from days 10 to 28 and a third peak at days 56–120 post immunization. The peaks in volunteer 50 s responses were not as dramatic as those observed in volunteer 53 s and 54 s.

The T_EMRA_ responses were generally lower in magnitude than T_EM_ responses in volunteer 53 s, essentially absent in volunteer 54 s, and substantially lower than T_EM_ responses in volunteer 50 s (Figure 2 B, D, & F). There were also some differences in the kinetics of the T_EM_ and T_EMRA_ responses (Figure 2 A–F).

### Differences in Kinetics of S. Typhi-specific HLA-E Restricted CD8+ T Cells

**Figure 3:**
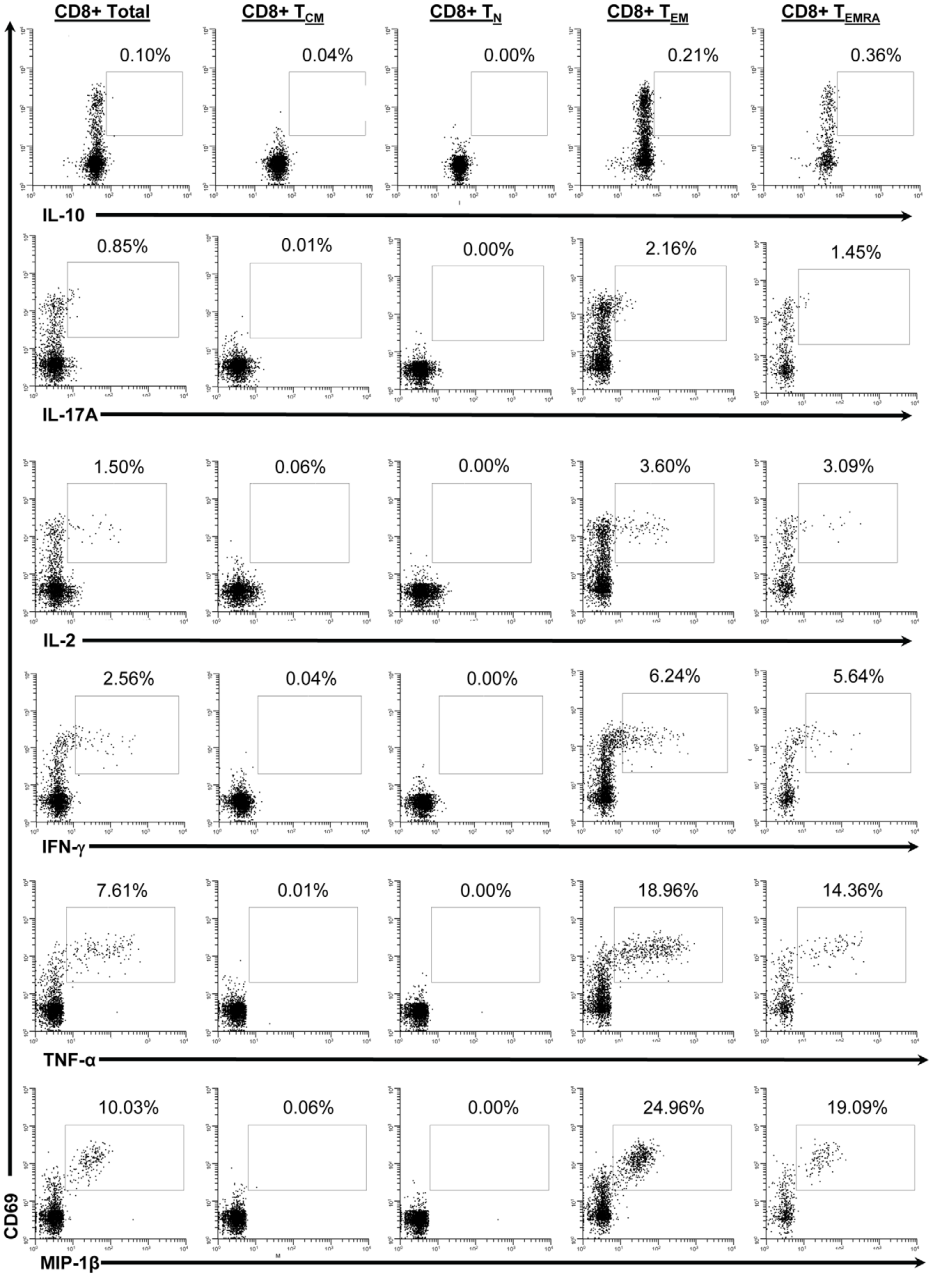
Detection of intracellular cytokines/chemokines produced by CD8+ T cell memory subsets in response to stimulation with *S.* Typhi-infected HLA-E restricted cells. Histograms from a representative volunteer (53 s at day 7 post infection) showing the production of 6 cytokines/chemokines by memory T cell subsets following stimulation with *S*. Typhi-infected AEH cells. CD69 (marker of recent activation) is displayed on the y-axis and each of the 6 cytokines/chemokines measured are displayed on the x-axes. Column 1 represents total CD8+ T cells, column 2 represents T_CM_, column 3 represents T_N_, column 4 represents T_EM_, and column 5 represents T_EMRA_. The percentage of positive cells is shown for the indicated region in each cytogram.

**Figure 4:**
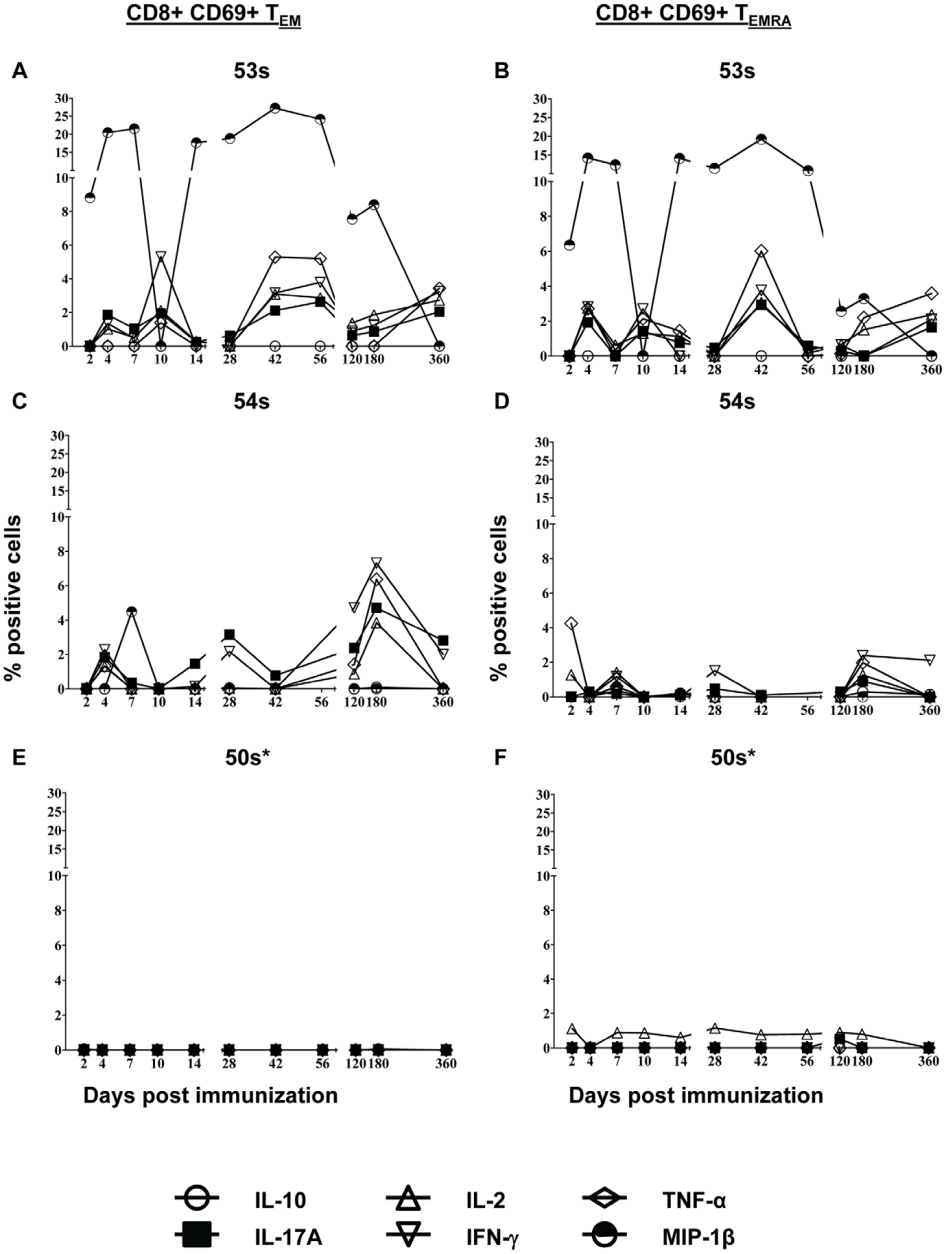
Kinetics of intracellular cytokine/chemokine production following stimulation of PBMC with *S*. Typhi-infected HLA-E restriced cells. Intracellular cytokine/chemokine production is shown as the percentage of positive cells. Responses are expressed as net values with day 0 subtracted to normalize for differences in base-line responses to *S*. Typhi in different volunteers. A) CD8+ T_EM_ cells for volunteer 53 s B) CD8+ T_EMRA_ cells for volunteer 53 s C) CD8+ T_EM_ cells for volunteer 54 s D) CD8+ T_EMRA_ cells for volunteer 54 s E) CD8+ T_EM_ cells for volunteer 50 s F) CD8+ T_EMRA_ cells for volunteer 50 s. Increases of >0.5% cytokine positive cells over uninfected targets were found to be statistically significant (P<0.01). * MIP-1b not measured.

Here we investigated whether IL-17A was produced following stimulation by HLA-E restricted CD8+ T cells, as well as defined the kinetics of this response. As described for PBMC stimulated with *S*. Typhi-infected autologous B-LCL, PBMC from immunized volunteers were stimulated with the *S*. Typhi-infected HLA-E restricted cell line (721.221.AEH), stained, and analyzed by multichromatic flow cytometry. The same gating strategy was applied (Figure S1). We found that T_EM_ and to a lesser extent T_EMRA_ subsets were the major sources of intracellular cytokine production (Figure 3). In contrast to stimulation with *S*. Typhi-infected autologous B-LCL, HLA-E restricted stimulation resulted in responses in 2 of the volunteers, 53 s and 54 s (Figure 4 and Figure S3). This is unlikely to be the result of technical difficulties. For all volunteers, including 50 s, there were equivalent viability and percentages of *S*. Typhi-infected autologous B-LCL and HLA-E restricted AEH cells (data not shown). With the exception of MIP-1β production by volunteer 53 s, the levels of detectable intracellular cytokines were lower in response to HLA-E restricted stimulation compared to autologous B-LCL stimulation (Figures 2 & 4). As seen following stimulation with *S*. Typhi-infected autologous B-LCL, MIP-1β production was the predominant response by volunteer 53 s (Figure 4 A & B). CD8+ T_EM_ from volunteer 54 s only produced MIP-1β at day 7 post immunization (Figure 4 C). TNF-α (5.29–6.37%) and IFN-γ (5.31–7.32%) had the highest percentage of cells with detectable intracellular cytokines, followed by IL-2 (2.87–3.85%) and IL-17A (2.64–4.71%) at peak time points (Figure 4). Although the kinetics were also bi- or tri-phasic, there were differences between the peak time-points following stimulation with *S*. Typhi-infected autologous B-LCL and HLA-E restricted cells (Figures 2 & 4). Volunteer 54 s showed a bi-phasic response to stimulation with *S*. Typhi-infected autologous B-LCL (Figure 2 C & D) but a tri-phasic response to *S*. Typhi-infected HLA-E restricted cells (Figure 4 C & D).

### Differences in the Kinetics of IL-17A Producing CD8+ T Cells in Response to Stimulation with S. Typhi-infected Autologous or HLA-E Restricted Cells

**Figure 5:**
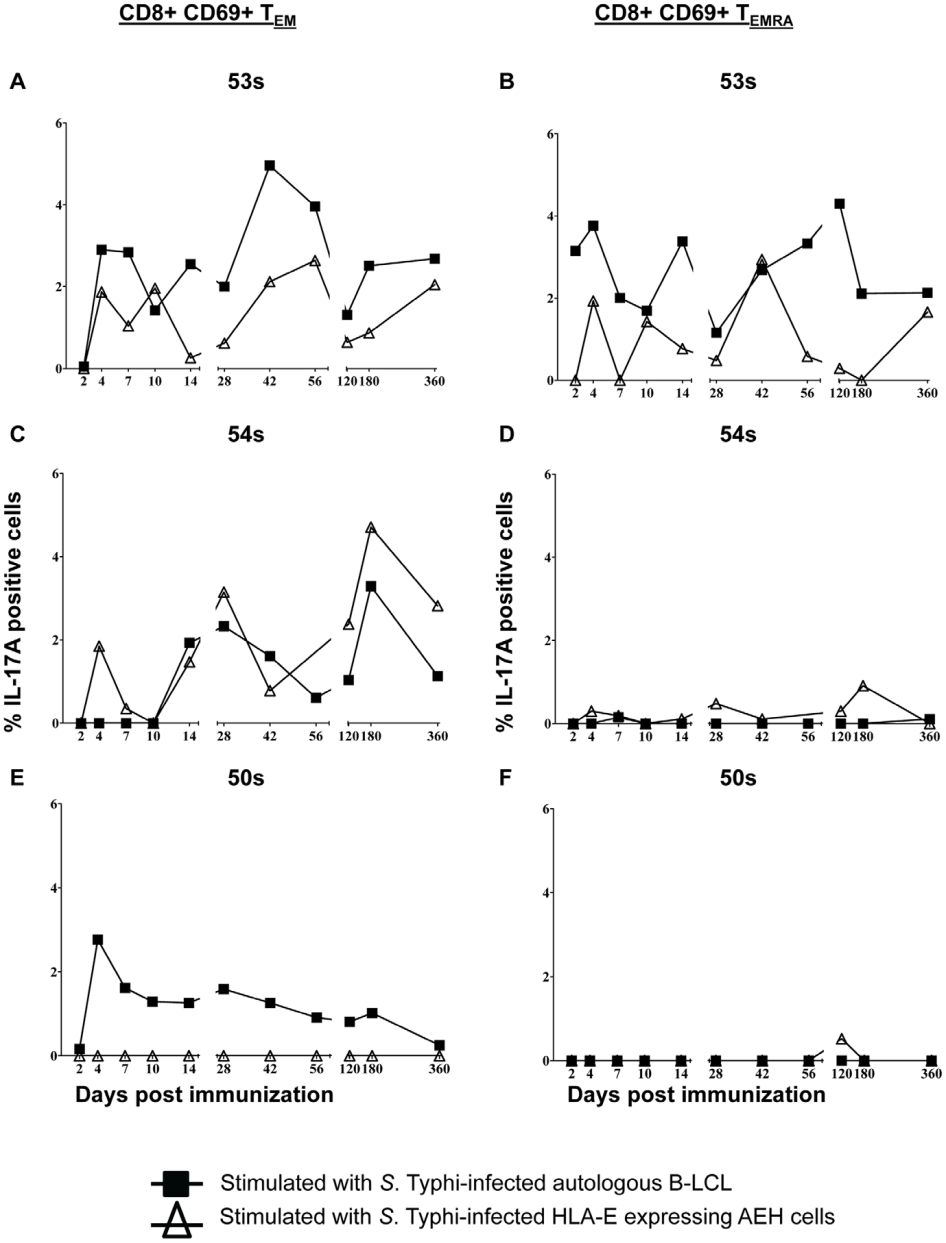
Kinetics of intracellular IL-17A production following stimulation with either *S*. Typhi-infected autologous B-LCL or HLA-E restricted cells. Intracellular cytokine/chemokine production is shown as the percentage of positive cells. Responses are expressed as net values with day 0 subtracted to normalize for differences in base-line responses to *S*. Typhi in different volunteers. Responses to *S*. Typhi-infected autologous B-LCL are indicated by a filled square (▪) and responses to *S.* Typhi-infected HLA-E restricted cells are indicated by open triangles (▵). A) CD8+ T_EM_ cells for volunteer 53 s B) CD8+ T_EMRA_ cells for volunteer 53 s C) CD8+ T_EM_ cells for volunteer 54 s D) CD8+ T_EMRA_ cells for volunteer 54 s E) CD8+ T_EM_ cells for volunteer 50 s F) CD8+ T_EMRA_ cells for volunteer 50 s. Increases of >0.5% cytokine positive cells over uninfected targets were found to be statistically significant (P<0.01).

This is, to our knowledge, the first description of CD8+ T cells producing IL-17A in response to *S*. Typhi. IL-17A was produced by all responding volunteers in response to stimulation with *S*. Typhi-infected autologous B-LCL (53 s, 54 s, and 50 s) and HLA-E restricted (53 s and 54 s) cells. T_EM_ were the predominant source of IL-17A, with intracellular IL-17A also detected in T_EMRA_ of volunteer 53 s (Figure 5 A & B). Only a small amount of intracellular IL-17A was detected in T_EMRA_ from volunteer 54 s in response to *S*. Typhi-infected HLA-E restricted cells on days 28 and 180 (Figure 5 D). The kinetics of IL-17A production were consistent with those of other cytokines measured (e.g, IL-2, IFN-γ, etc) and demonstrated bi- or tri-phasic responses that differed according to the *S.* Typhi-infected targets used in the stimulation (Figure 5).

### S. Typhi-specific CD8+ T Cells are Primarily Multifunctional

**Figure 6:**
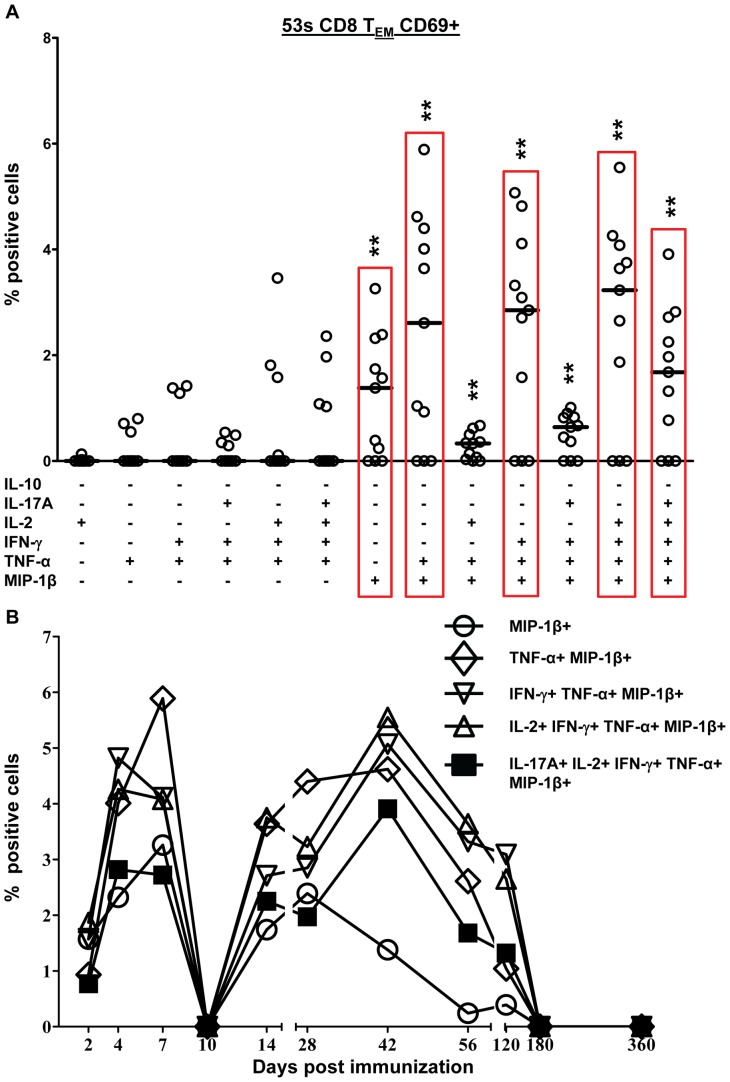
Multifunctional CD8+ T_EM_ responses to *S*. Typhi-infected autologous B-LCL. A) Scatter plot showing all combinations of the 6 cytokines/chemokines measured that were positive at one or more time points for representative volunteer 53 s. Increases of >0.5% positive cells over uninfected targets were statistically significant (P<0.01). Each point represents a single time-point and the median value is denoted by a horizontal bar. The cyokine/chemokine combinations with the top 5 median values are indicated by red rectangles. Median values of the subsets of multifunctional cells that show a significant difference (**P<0.01), when compared to the subsets without asterisks, are indicated. B) Kinetics of the top 5 populations (as determined by median value) over time.

**Figure 7:**
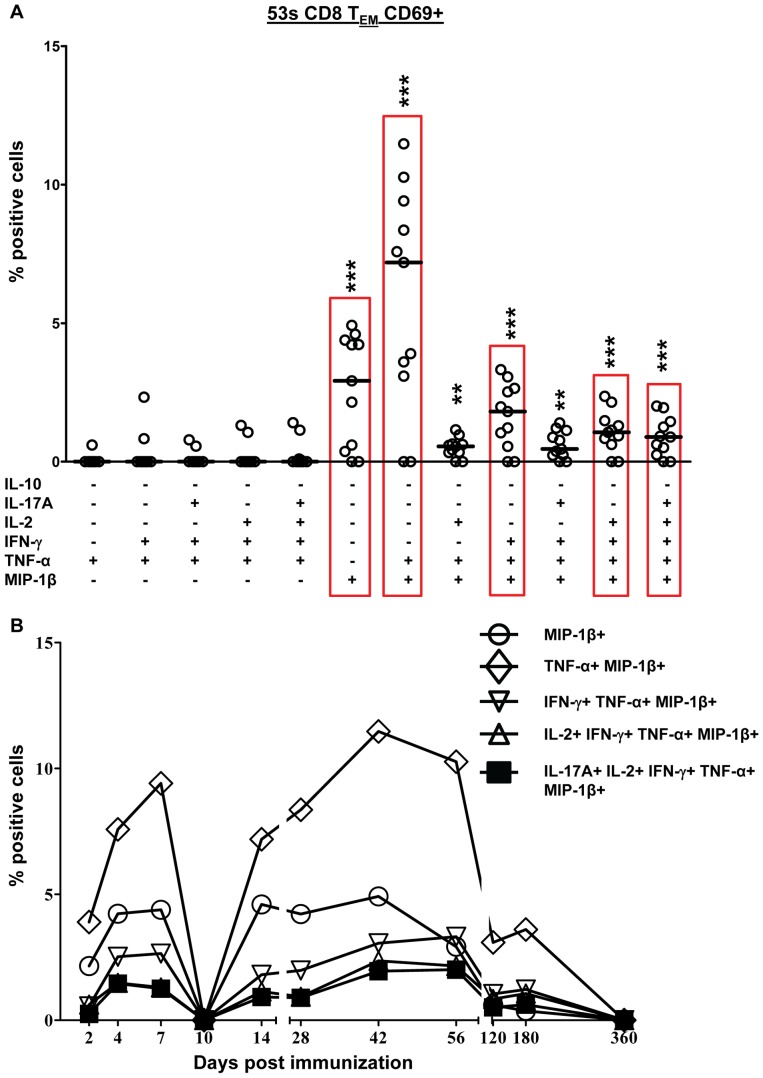
Multifunctional CD8+ T_EM_ responses to *S*. Typhi-infected HLA-E restricted cells. A) Scatter plot showing all combinations of the 6 cytokines/chemokines measured that were positive at one or more time points for representative volunteer 53 s. Increases of >0.5% positive cells over uninfected targets were statistically significant. Each point represents a single time-point and the median value is denoted by a horizontal bar. The cyokine/chemokine combinations with the top 5 median values are indicated by red rectangles. Median values of the subsets of multifunctional cells that show a significant difference (**P<0.01, ***P<0.001), when compared to the subsets without asterisks, are indicated. B) Kinetics of the top 5 populations (as determined by median value) over time.

Because recent reports suggested that multifunctional T cells are likely to play a prominent role in protection from infection [25], [26], [27], we next investigated the multifunctionality of CD8+ T cell responses to Ty21a. Using the FCOM function of WinList (Verity software) we analyzed all possible combinations (64 total) of the 6 cytokines/chemokines measured (IL-10, IL-17A, IL-2, IFN-γ, TNF-α, and MIP-1β) at each time-point. Only combinations with at least 1 time-point ≥0.5% of CD8+ T_EM_ or T_EMRA_ cytokine positive cells are shown. This percentage represents a minimum of ∼10–50 events for T_EMRA_ and T_EM_ respectively. Results from a representative volunteer (53 s) are shown in response to stimulation with *S*. Typhi-infected autologous cells (Figure 6) and *S*. Typhi-infected HLA-E restricted cells (Figure 7). Data from the other responding volunteers are shown in Figures S3,S4,S5. Due to the complexity of the data (64 combinations for each of 12 time-points) we determined the median value for each combination that was ≥0.5% based on all time-points (Figures 6A, 7A, S3A, S4A, S5A). Each combination is shown on the X-axis with the values from each time-point (in % positive cells) on the Y-axis. The median value was determined for each combination and the kinetics of the top 5 combinations are shown (Figures 6B, 7B). Multifunctional responses predominated over single cytokine producing cells in all volunteers (Figures 6 and 7, and S3,S4,S5). As demonstrated in Figure 6, 4 of the top 5 combinations identified were multifunctional for volunteer 53 s in response to autologous stimulation. All of the top 5 combinations for volunteer 53 s produced MIP-1β (either singly or in combination with other cytokines). The top 5 combinations for volunteer 53 s were the same for both autologous and HLA-E restricted stimulation (Figures 6 & 7). In contrast, in volunteer 54 s the top combinations differed depending on the type of stimulation (Figures S3 and S4). Volunteer 50 s did not respond to HLA-E restricted stimulation.

### IL-17A Producing Cells are Multifunctional and co-produce IL-2, IFN-γ, TNF-α, and/or MIP-1β

**Figure 8:**
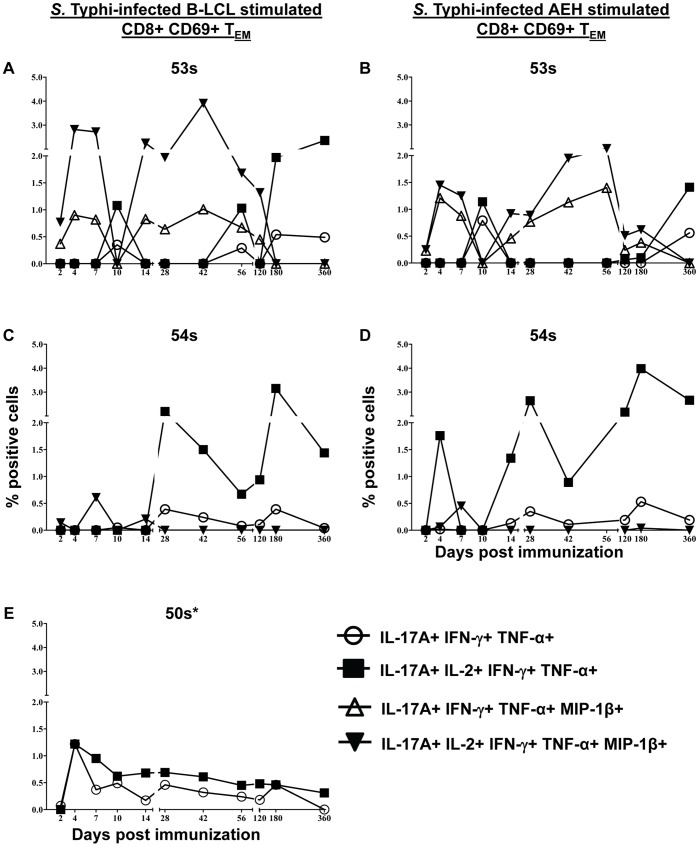
Multifunctional CD8+ T_EM_ IL-17A+ populations. Kinetics of multifunctional IL-17A+ populations (if net >0.5%) following stimulation with either *S*. Typhi-infected autologous B-LCL (A, C, & E) or HLA-E restricted cells (B & D). Net increases of >0.5% cytokine positive cells over uninfected targets were found to be statistically significant (P<0.01). 50 s had no response to HLA-E restricted stimulation. * MIP-1b was not measured.

As shown above, IL-17A production was detected in all of the responding volunteers following stimulation with both autologous and HLA-E restricted cells (Figures 2, 4, and S3,S4,S5). To further characterize the IL-17A producing cells, we studied their multifunctional characteristics in further detail. We found that virtually all of the IL-17A producing cells were multifunctional, co-producing IL-2, IFN-γ, TNF-α, and/or MIP-1β (Figure 8). All IL-17A+ populations were IL-10-. We identified 4 IL-17A+ populations: IL-17A+ IL-2+ IFN-γ+ TNF-α+ MIP-1β- (“quadruple positive”), IL-17A+ IL-2- IFN-γ+ TNF-α+ MIP-1β- (“triple positive”), IL-17A+ IL-2+ IFN-γ+ TNF**-**α+ MIP-1β+ (“quintuple positive”), and IL-17A+ IL-2- IFN-γ+ TNF-α+ MIP-1β+ (“quadruple positive”). IL-17A production by volunteer 53 s was predominantly by quintuple-positive cells co-producing IL-17A, IL-2, IFN-γ, TNF-α, and MIP-1β at most time points. The peak of the quintuple-positive cells was on day 42 post immunization following stimulation with *S*. Typhi-infected autologous B-LCL (3.91% of CD8+ CD69+ T_EM_) and day 56 post immunization following stimulation with *S*. Typhi-infected HLA-E restricted cells (2.01% of CD8+ CD69+ T_EM_) (Figure 8 A & B). Quadruple-positive (IL-17A+ IL-2- IFN-γ+ TNF-α+ MIP-1β+) cells were also present at lower percentages with similar kinetics. Additionally, at day 10 post immunization there was no MIP-1β; however production of IL-17A+ IL-2+ IFN-γ+ TNF-α+ MIP-1β- and IL-17A+ IL-2- IFN-γ+ TNF-α+ MIP-1β- populations were identified (although at lower levels) (Figure 8 A & B). Volunteer 54 s did not show any MIP-1β production except on day 7 post immunization (Figure 2C and Figure 4C). IL-17A+ populations reflected this with the predominant populations being quadruple positive (IL-17A+ IL-2+ IFN-γ+ TNF-α+ MIP-1β-) followed by triple positive (IL-17A+ IL-2- IFN-γ+ TNF-α+ MIP-1β-) T_EM_ cells at all time-points except at day 7 post immunization (Figure 8 C & D). MIP-1β was not measured in volunteer 50 s so only 2 populations were identified. These populations varied only in their production of IL-2 (Figure 8 E).

### Unsupervised Analysis Using Flow Cytometry Clustering without K (FLOCK) Confirms the Multifunctionality of S. Typhi-specific CD8+ IL-17A+ T Cells

**Figure 9:**
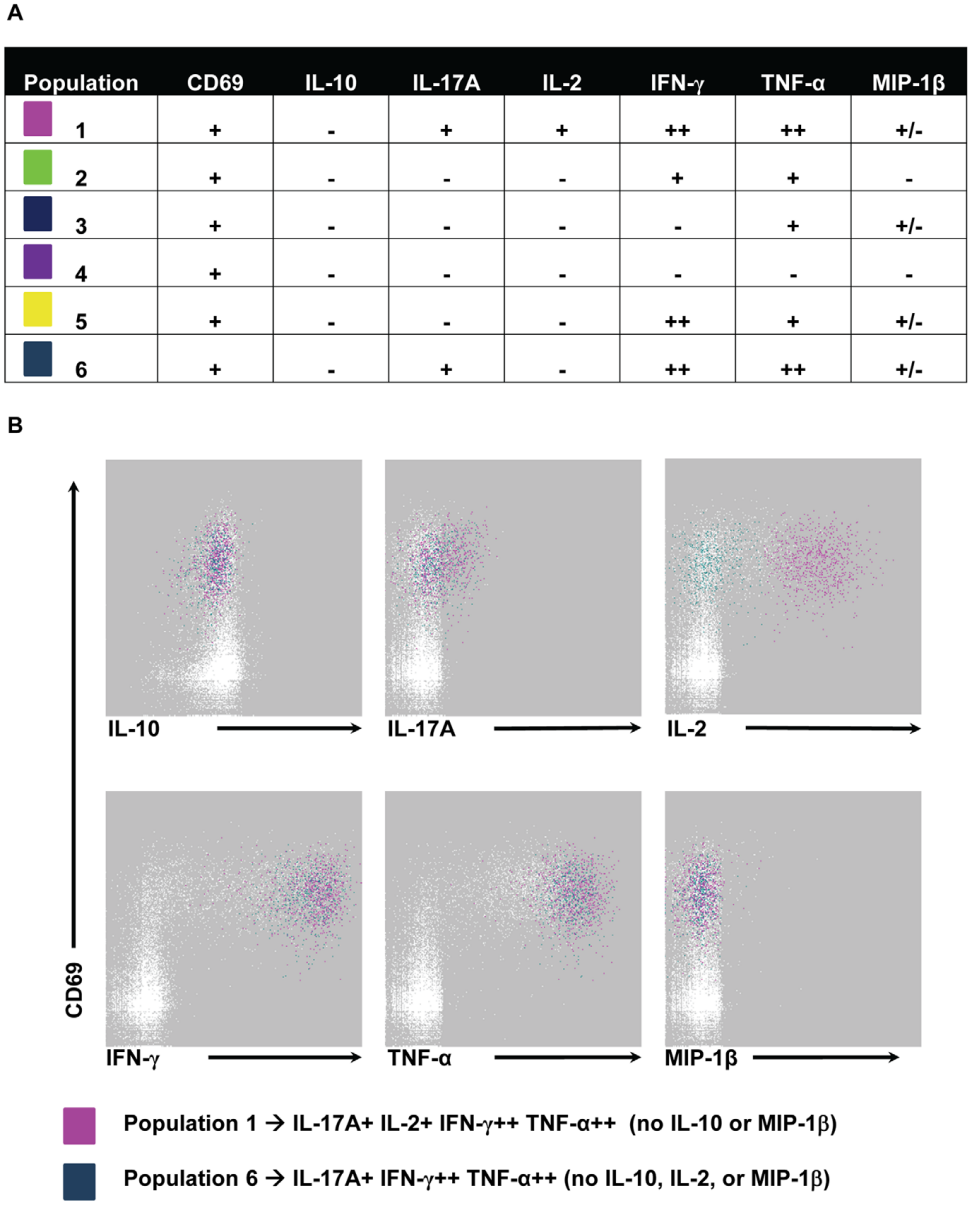
CD69+ populations identified by unsupervised FLOCK analyses. CD3+ CD8+ T_EM_ events following *S*. Typhi-infected autologous B-LCL stimulation were uploaded to ImmPort for FLOCK analyses. A) Cytokine/chemokine production patterns of the 6 CD69+ populations identified by FLOCK. B) Histograms showing FLOCK of representative volunteer 53 s at day 10 post immunization with the IL-17A+ populations highlighted (population 1- pink and population 6- aqua).

To further examine the multifunctional IL-17A+ populations identified by conventional, user-guided, flow cytometry analyses, we used the novel unsupervised analysis program FLOCK. FLOCK uses computational methods to determine the number of unique populations in multidimensional flow cytometry data [36]. A subset of time-points (days 0, 7, 10, and 42 post immunization) for volunteers 53 s and 54 s following stimulation with *S.* Typhi-infected B-LCL were analyzed. The same data that were analyzed for intracellular detection of IL-10, IL-17A, IL-2, IFN-γ, TNF-α, and MIP-1β by conventional, user-guided methods (Figures 1, 6, and S3) were analyzed by FLOCK. Prior to FLOCK analyses, gating was performed as described in Figure S1 to select CD3+ CD8+ T_EM_ events. Data for the 4 selected time-points for each volunteer were uploaded to the ImmPort website (http://immport.niaid.nih.gov) and FLOCK analyses performed. The number of unique populations varied at different time-points and between volunteers (9–29 individual populations). In order to compare data across time-points and between volunteers, a cross-sample analysis was performed. In this cross sample analysis, the populations identified in a single sample (volunteer 53 s day 10 post infection following stimulation with *S*. Typhi-infected B-LCL) were applied to all samples. Six unique CD69+ (i.e., recently activated) populations were identified (Figure 9 A). All 6 populations were negative for IL-10 production. MIP-1β production was dependent on the time-point and volunteer. In other words, at those time-points for volunteer 53 s at which intracellular MIP-1β was detected (days 7 and 42 post immunization), MIP-1β was produced by populations 1, 3, 5, and 6. However, for volunteer 54 s, only populations 3 and 5 produced MIP-1β on day 7 post immunization. Population 4 was negative for all cytokines/chemokines measured. Populations 1 and 6 were IL-17A+ (Figure 9). The IL-17A+ populations identified were both multifunctional co-producing IL-2, IFN-γ, TNF-α, and/or MIP-1β (Figure 9). Population 1 was IL-10- IL-17A+ IL-2+ IFN-γ+ TNF-α+ MIP-1β+/−, while population 6 was IL-10- IL-17A+ IL-2- IFN-γ+ TNF-α+ MIP-1β+/− (Figure 9). Figure 9 depicts a representative sample which did not produce MIP-1β (volunteer 53 s day 10 post immunization). The IL-17A+ populations that were identified differed in their production of IL-2.

In addition to identifying multifunctional cells, FLOCK analysis distinguished bright (++) and dim (+) populations of IFN-γ and TNF-α based on mean fluorescence intensity (MFI) (Figure 9 A). Brighter intensity of staining indicates higher intracellular cytokine content. Populations 1, 5, and 6 stained brightly for IFN-γ, while populations 1 and 6 stained brightly for TNF-α (Figure 9). These data from unsupervised analysis indicate that IL-17A-producing cells produce high levels of both IFN-γ and TNF-α.

**Figure 10:**
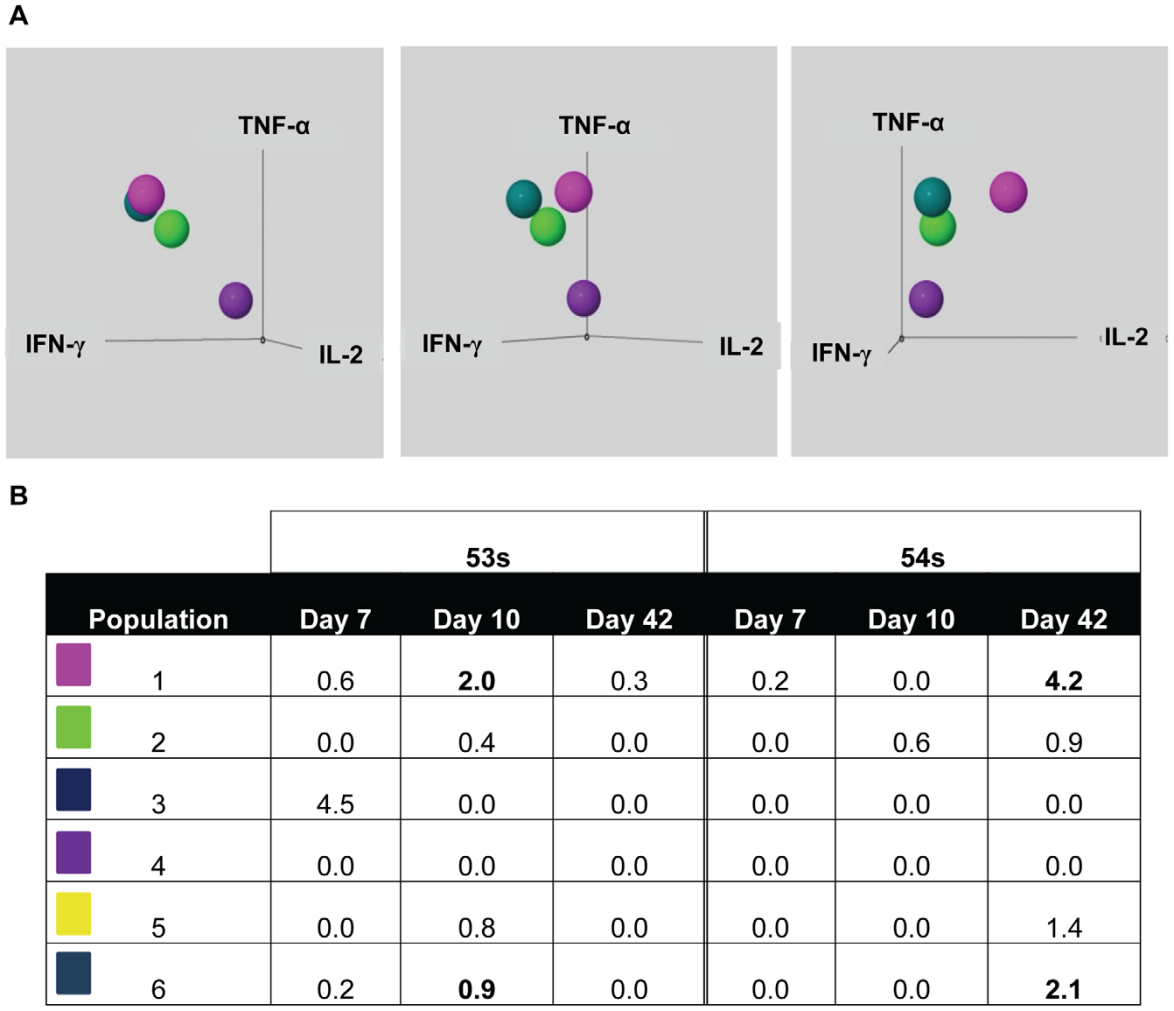
Three-dimensional representation of FLOCK data and percentages for each of the CD69+ populations at 3 time-points. A) Populations 1 (pink), 2 (lime green), 4 (purple), and 6 (aqua) shown in 3-dimensions. IL-2 is shown on the x-axis, IFN-γ on the y-axis, and TNF-α on the z-axis. B) Cross-sample analysis results showing net (day 0 subtracted) percentages of CD8+ T_EM_ for each of the CD69+ populations at days 7, 10, and 42 post immunization for volunteers 53 s and 54 s. Peak responses for each population are bolded.

Figure 10A shows 3-dimensional representation of IL-2 (x-axis), IFN-γ (y-axis), and TNF-α (z-axis) for 4 of the CD69+ populations (populations 1, 2, 4, and 6). Each panel shows an approximately 30° rotation around the z-axis. Population 4 (purple) is negative for all measured cytokines, population 2 (lime green) is IFN-γ and TNF-α dim, while populations 1 (pink) and 6 (aqua) are IFN-γ and TNF-α bright. Populations 1 and 6 are both IL-17A+ but differ in their production of IL-2 as described above (Figures 9A and B and 10A).

The net percentage of positive cells for each population at the 3 time-points, as determined by cross-sample analysis, is shown in Figure 10B. IL-17A+ multifunctional cells were identified at days 7, 10, and 42 post immunization for volunteer 53 s with a peak at day 10 post immunization (Figure 10B). Volunteer 54 s had low levels of IL-17A+ multifunctional cells on day 7 post immunization, but the predominant response was at day 42 post immunization which correlates with the later peak cytokine production previously demonstrated for volunteer 54 s (Figure 2C).

## Discussion

Here we demonstrate the presence and persistence of multifunctional IL-17A producing *S*. Typhi-specific CD8+ T cells in response to both *S.* Typhi-infected autologous B-LCL and HLA-E restricted stimulation. Multifunctional IL-17A+ cells demonstrated multiphasic kinetics and were still detectable one year after immunization. We identified quadruple and quintuple positive CD8+ T_EM_ and T_EMRA_ IL-17A producing cells that co-produce pro-inflammatory cytokines/chemokines IL-2, IFN-γ, TNF-α, and/or MIP-1β but not IL-10. These multifunctional populations were confirmed using unsupervised flow cytometric analysis with FLOCK.

IL-17 has been increasingly implicated in host responses against intracellular pathogens [19]. Specifically, the importance of IL-17 in mucosal immune responses to intracellular enteric pathogens has been demonstrated in animal models [20], [21]. It was shown that depletion of Th17 cells during simian immunodeficiency virus (SIV) infection results in increased dissemination of *Salmonella* Typhimurium from the gut [20]. Additionally, antigen-specific IL-17A+ cells were identified in response to *Salmonella* Enteriditis infection and IL-17A knockout mice had an elevated bacterial burden in the liver and spleen as compared to wild-type mice [37]. Thus, it was of great importance to initiate studies to evaluate whether IL-17A might play a role in protection from *S.* Typhi. Because the gastrointestinal mucosa is the first point of contact for *S.* Typhi, mucosal immune responses are likely to play an important role in protection. This is to our knowledge, the first report of IL-17A production in response to *S*. Typhi, both to *S.* Typhi-infected autologous targets, as well as HLA-E restricted stimulation. The presence of *S*. Typhi-specific CD8+ T cells producing IL-17A suggests that these cells may play a role in the mucosal response. CD8+ T cells with characteristics of the Th17 lineage, Tc17, have been described in the setting of chronic hepatitis C virus infection [25]; however, in these studies, stimulation with phorbol myristate acetate (PMA) and ionomycin was used rather than specific antigen. Thus, the results presented in this manuscript are, to our knowledge, the first demonstration in humans of the presence of CD8^+^ Tc17 cells responsive to antigens from infectious agents.

Multifunctional T cells, *i.e*., those producing 2 or more cytokines simultaneously, have been shown to produce higher levels of individual cytokines, have enhanced function, and are more likely to correlate with protection from disease, when compared to single cytokine producing cells [29], [30], [31]. Although T cells co-producing IL-17A with IFN-γ or TNF-α in response to PMA/ionomycin stimulation have been identified [38], [39], to our knowledge, this is the first study to examine the simultaneous production of IL-17A with 5 other cytokines/chemokines, which play key roles in inflammatory processes, in a human-pathogen specific system. Of note, unsupervised flow cytometric FLOCK analysis showed higher levels of IFN-γ and TNF-α being produced by IL-17A-producing multifunctional cells (i.e., multifunctional IL-17A+ populations are bright for IFN-γ and TNF-α). In addition, we observed that IL-17A was produced exclusively by multifunctional cells and that the predominant IL-17A+ responses were IL-17A+ IL-2+ IFN-γ+ TNF-α+ MIP-1β+/− as assessed by both supervised (FCOM) and unsupervised (FLOCK) flow cytometric analyses. The co-production of IL-17A with cytokines typically associated with Tc1 responses, suggest that the Tc17 lineage is not as distinct in humans as *in vitro* and animal studies have previously suggested.

Multiphasic kinetics have previously been demonstrated in response to Ty21a immunization following stimulation in an HLA-E restricted manner [13]. Here we confirm and extend these findings by showing that multiphasic kinetics are also typical of responses to autologous stimulation with B-LCL. Interestingly, despite their bi- or tri-phasic nature, the kinetics of responses to autologous stimulation and HLA-E restricted stimulation differed. HLA-E is a non-classical major histocompatibility (MHC) molecule which is highly conserved [40] and it is expressed ubiquitously on PBMC (at various levels depending on the individual cell types) in combination with classical MHC molecules [41]. Given the differences in kinetics between autologous and HLA-E restricted stimulation, as well as the fact that volunteer 50 s showed responses to autologous stimulation but not to HLA-E restricted stimulation, our results clearly indicate that while HLA-E restricted responses may contribute to the responses seen when PBMC were stimulated with *S.* Typhi-infected autologous B-LCL, they represent only a portion of these responses. It will be important to confirm these findings using different targets such as autologous blasts. However, these data are supported by the fact that in previous studies, monoclonal antibodies against HLA-E were only able to partially block CTL activity against *S*. Typhi-infected targets, while monoclonal antibody against a pan-MHC class I marker (W6/32) showed almost complete abrogation of CTL against *S*. Typhi-infected targets [9]. Moreover, the observations that at different times after immunization there are considerable differences in the effector subsets that respond to autologous and HLA-E restricted *S.* Typhi-infected cells raises the likely possibility that various effector cells might contribute differently to the host defense at various times after exposure. These observations also point to the importance of the timing of specimen collection following immunization and its impact on the magnitude of effector immune responses which can be measured. Finally, it is important to mention that these data confirm and extend previous observations on the longevity of CMI responses (at least 1 year) elicited following immunization with Ty21a and other attenuated typhoid vaccine candidates [12], [13].

The significance of the multiphasic responses observed is not entirely clear; however, it is possible that the decline in the levels of cytokine producing *S*. Typhi-specific CD8+ T cells at various times could represent these cells homing cells to the gut. Previous studies have, in fact, shown expression of gut-homing molecules on *S*. Typhi-specific CD8+ T cells [12], [17]. While these multiphasic responses have previously been described for HLA-E restricted responses [13], this is the first report of multiphasic kinetics in response to autologous stimulation over the course of 1 year post-immunization with the Ty21a typhoid vaccine. As has previously been shown, the responses following HLA-E restricted stimulation peaked as early as 4–7 days post immunization which is earlier than typically expected for CMI responses [13]. Interestingly, in 2 of the 3 responding volunteers (50 s and 53 s), a peak as early as days 4–7 post immunization was observed in response to autologous stimulation as well. In summary, multifunctional IL-17A+ CD8+ T cells display multiphasic kinetics in response to both classical and non-classical MHC restricted stimulation. These responses persist at least 1 year after immunization. Future studies in which the presence of these multifunctional Tc17 responses are evaluated in the context of clinical outcome following challenge of volunteers with wild-type *S*. Typhi will provide key information to assess the role of multifunctional cells producing high levels of IL-17A and other cytokines in protection from infection. These studies are likely to contribute to accelerate the development of much needed novel typhoid vaccines.

## Supporting Information

Figure S1 **Gating strategy used for flow cytometry analyses.** Lymphocytes were gated based on forward and side scatter, followed by doublet exclusion. Live (ViViD negative) CD14- CD19- CD3+ T cells were then selected. CD8+ CD4- cells were further divided into memory subsets based on CD62L and CD45RA expression. Naïve T cells (T_N_; CD62L+ CD45RA+), T central memory cells (T_CM_; CD62L+ CD45RA-), T effector memory (T_EM_; CD62L- CD45RA-), and T effector memory CD45RA+ (T_EMRA_; CD62L- CD45RA+)
(TIF)

Figure S2 **Kinetics of cytokine/chemokine production following stimulation of PBMC with** ***S*** **. Typhi-infected autologous B-LCL (non-responders and a non-immunized control).** Responses are expressed as net values with day 0 subtracted to normalize for differences in base-line responses to *S*. Typhi in different volunteers. A) CD8+ T_EM_ cells for volunteer 48 s B) CD8+ T_EMRA_ cells for volunteer 48 s C) CD8+ T_EM_ cells for volunteer 52 s D) CD8+ T_EMRA_ cells for volunteer 52 s E) CD8+ T_EM_ cells for volunteer 196 s F) CD8+ T_EMRA_ cells for volunteer 196 s * MIP-1b not measured
(TIF)

Figure S3 **Multifunctional CD8+ T_EM_ responses to** ***S*** **. Typhi-infected autologous B-LCL.** A) Scatter plot showing all combinations of the 6 cytokines/chemokines measured that were positive at one or more time points for volunteer 54 s. Increases of >0.5% cytokine positive cells over uninfected targets were found to be statistically significant (P<0.01). Each point represents a single time-point and the median value is denoted by a horizontal bar. The cyokine/chemokine combinations with the top 2 median values are indicated by red squares. Median values of the subsets of multifunctional cells that show a significant difference (*P<0.05, **P<0.01), when compared to the subsets without asterisks, are indicated. B) Kinetics of the top 2 populations (as determined by median value) over time.
(TIF)

Figure S4 **Multifunctional CD8+ T_EM_ responses to** ***S*** **. Typhi-infected HLA-E restricted cells.** A) Scatter plot showing all combinations of the 6 cytokines/chemokines measured that were positive at one or more time points for volunteer 54 s. Net increases of >0.5% cytokine positive cells over uninfected targets were found to be statistically significant (P<0.01). Each point represents a single time-point and the median value is denoted by a horizontal bar. The cyokine/chemokine combinations with the top 2 median values are indicated by red rectangles. Median values of the subsets of multifunctional cells that show a significant difference (*P<0.05, **P<0.01), when compared to the subsets without asterisks, are indicated. B) Kinetics of the top 2 populations (as determined by median value) over time.
(TIF)

Figure S5 **Multifunctional CD8+ T_EM_ responses to** ***S*** **. Typhi-infected autologous B-LCL.** A) Scatter plot showing all combinations of the 5 cytokines/chemokines measured (MIP-1β was not measured) that were positive at one or more time points for volunteer 50 s. Net increases of >0.5% cytokine positive cells over uninfected targets were found to be statistically significant (P<0.01). Each point represents a single time-point and the median value is denoted by a horizontal bar. The cyokine/chemokine combinations with the top 3 median values are indicated by red rectangles. Median values of the subsets of multifunctional cells that show a significant difference (*P<0.05, **P<0.01, ***P≤0.001), when compared to the subsets without asterisks, are indicated. B) Kinetics of the top 3 populations (as determined by median value) over time.
(TIF)
