# Supplementary figures and images for: Heterogeneity of Multifunctional IL-17A Producing S. Typhi-Specific CD8+ T Cells in Volunteers following Ty21a Typhoid Immunization
Source: PLoS One. 2012 Jun 5;7(6):e38408. doi: 10.1371/journal.pone.0038408 (PMC3367967; doi:10.1371/journal.pone.0038408)

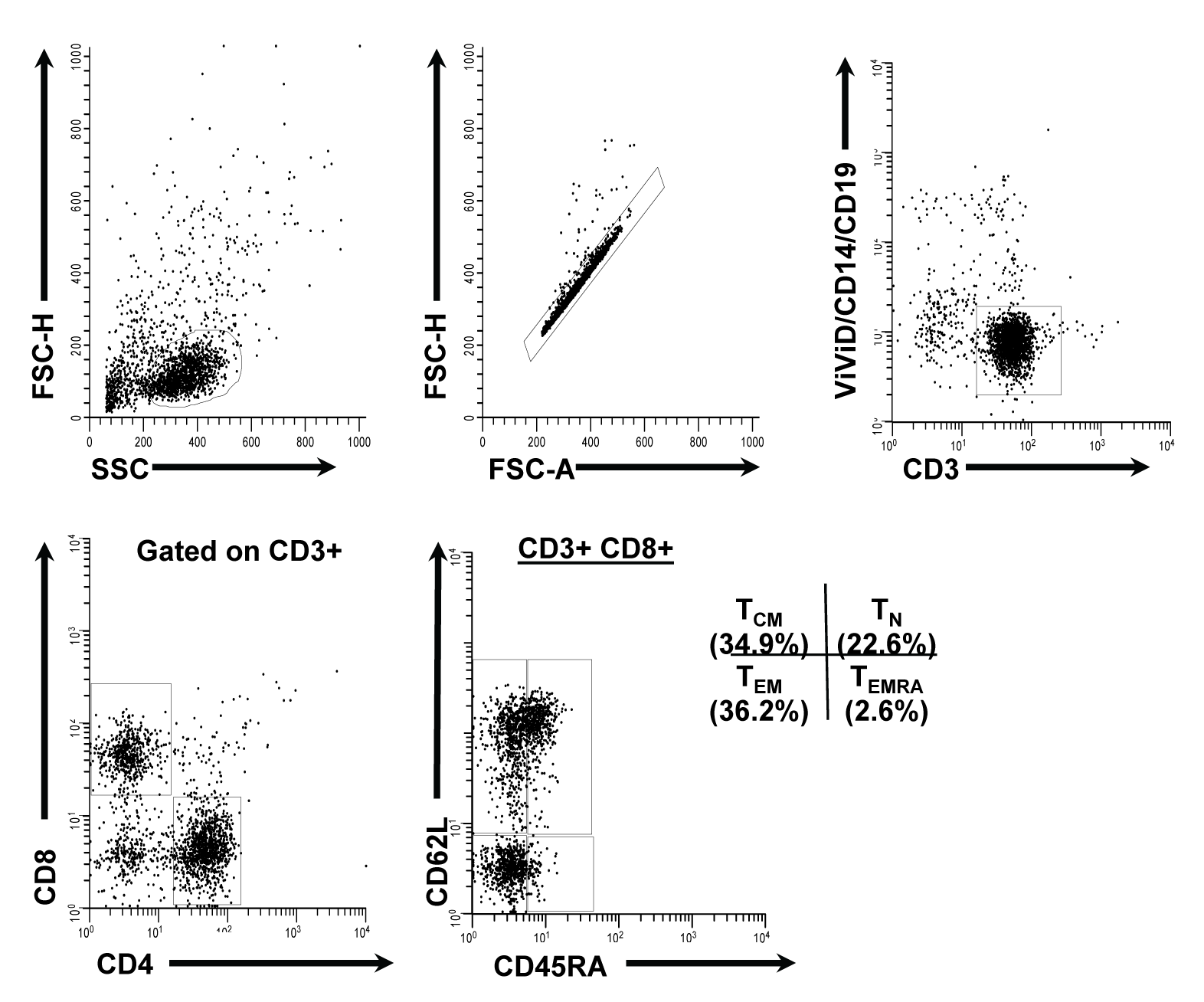

Supplement: Figure S1 — Gating strategy used for flow cytometry analyses. Lymphocytes were gated based on forward and side scatter, followed by doublet exclusion. Live (ViViD negative) CD14- CD19- CD3+ T cells were then selected. CD8+ CD4- cells were further divided into memory subsets based on CD62L and CD45RA expression. Naïve T cells (TN; CD62L+ CD45RA+), T central memory cells (TCM; CD62L+ CD45RA-), T effector memory (TEM; CD62L- CD45RA-), and T effector memory CD45RA+ (TEMRA; CD62L- CD45RA+) (TIF) [file pone.0038408.s001.tif]

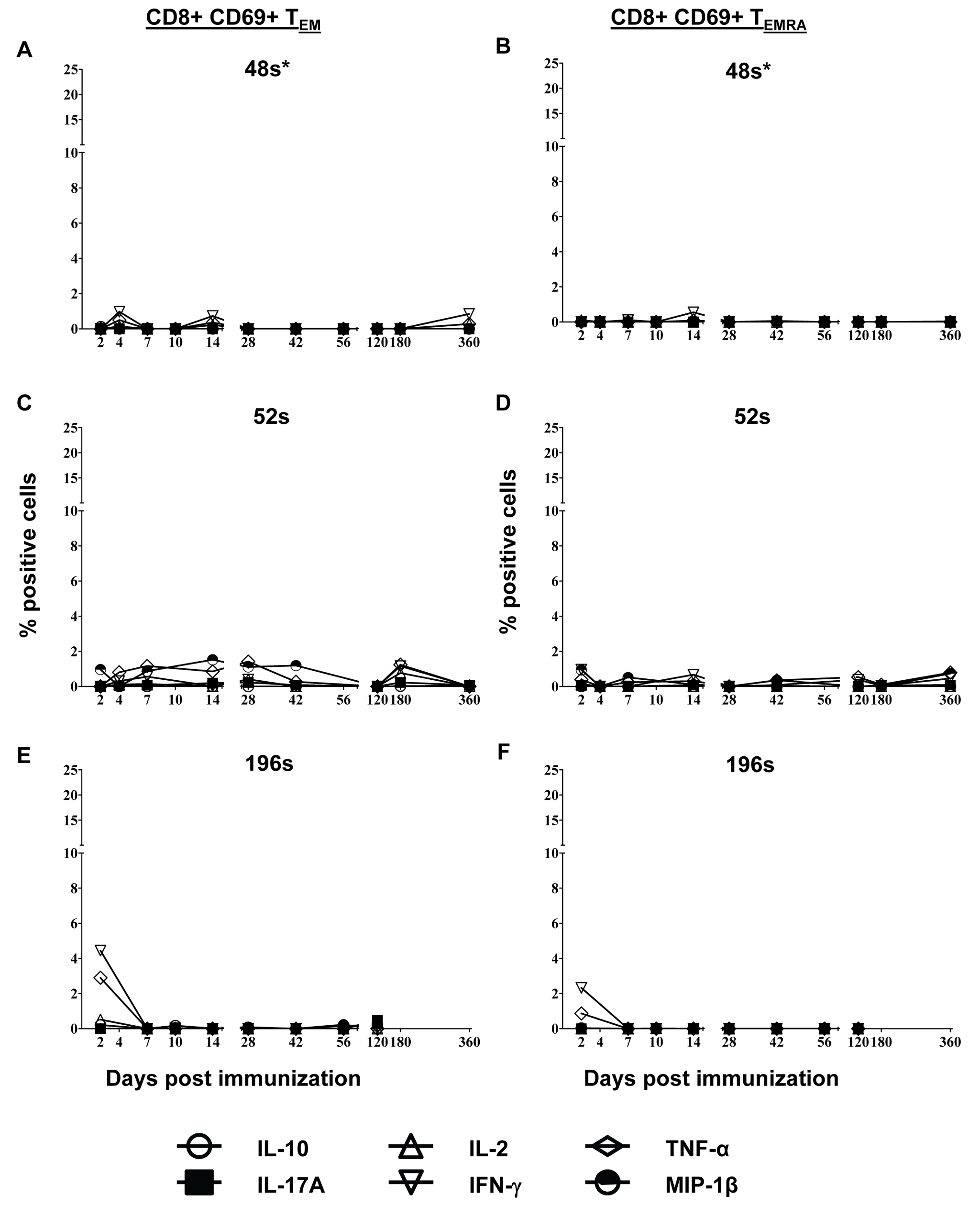

Supplement: Figure S2 — Kinetics of cytokine/chemokine production following stimulation of PBMC with S . Typhi-infected autologous B-LCL (non-responders and a non-immunized control). Responses are expressed as net values with day 0 subtracted to normalize for differences in base-line responses to S. Typhi in different volunteers. A) CD8+ TEM cells for volunteer 48 s B) CD8+ TEMRA cells for volunteer 48 s C) CD8+ TEM cells for volunteer 52 s D) CD8+ TEMRA cells for volunteer 52 s E) CD8+ TEM cells for volunteer 196 s F) CD8+ TEMRA cells for volunteer 196 s * MIP-1b not measured (TIF) [file pone.0038408.s002.tif]

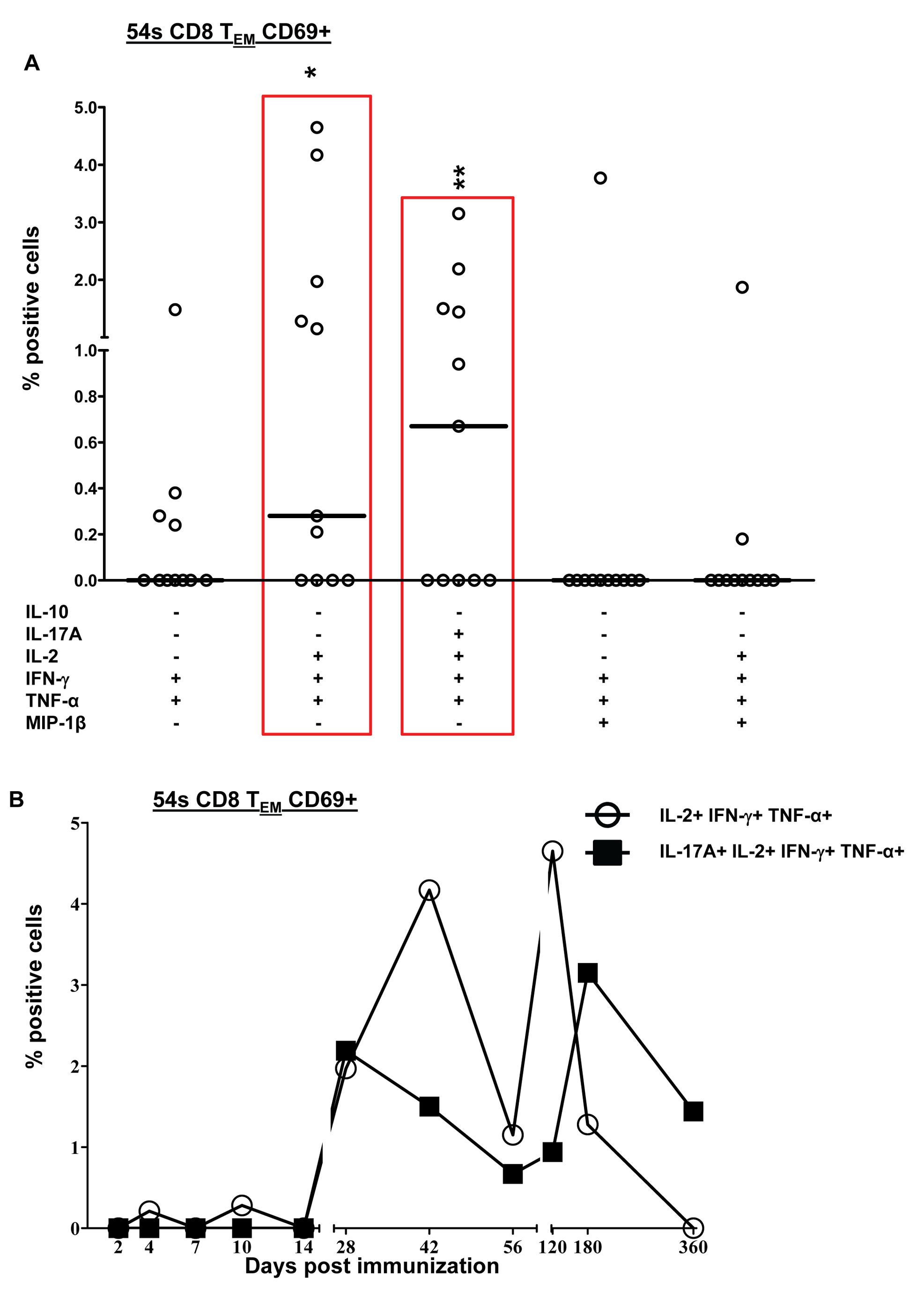

Supplement: Figure S3 — Multifunctional CD8+ TEM responses to S . Typhi-infected autologous B-LCL. A) Scatter plot showing all combinations of the 6 cytokines/chemokines measured that were positive at one or more time points for volunteer 54 s. Increases of >0.5% cytokine positive cells over uninfected targets were found to be statistically significant (P<0.01). Each point represents a single time-point and the median value is denoted by a horizontal bar. The cyokine/chemokine combinations with the top 2 median values are indicated by red squares. Median values of the subsets of multifunctional cells that show a significant difference (*P<0.05, **P<0.01), when compared to the subsets without asterisks, are indicated. B) Kinetics of the top 2 populations (as determined by median value) over time. (TIF) [file pone.0038408.s003.tif]

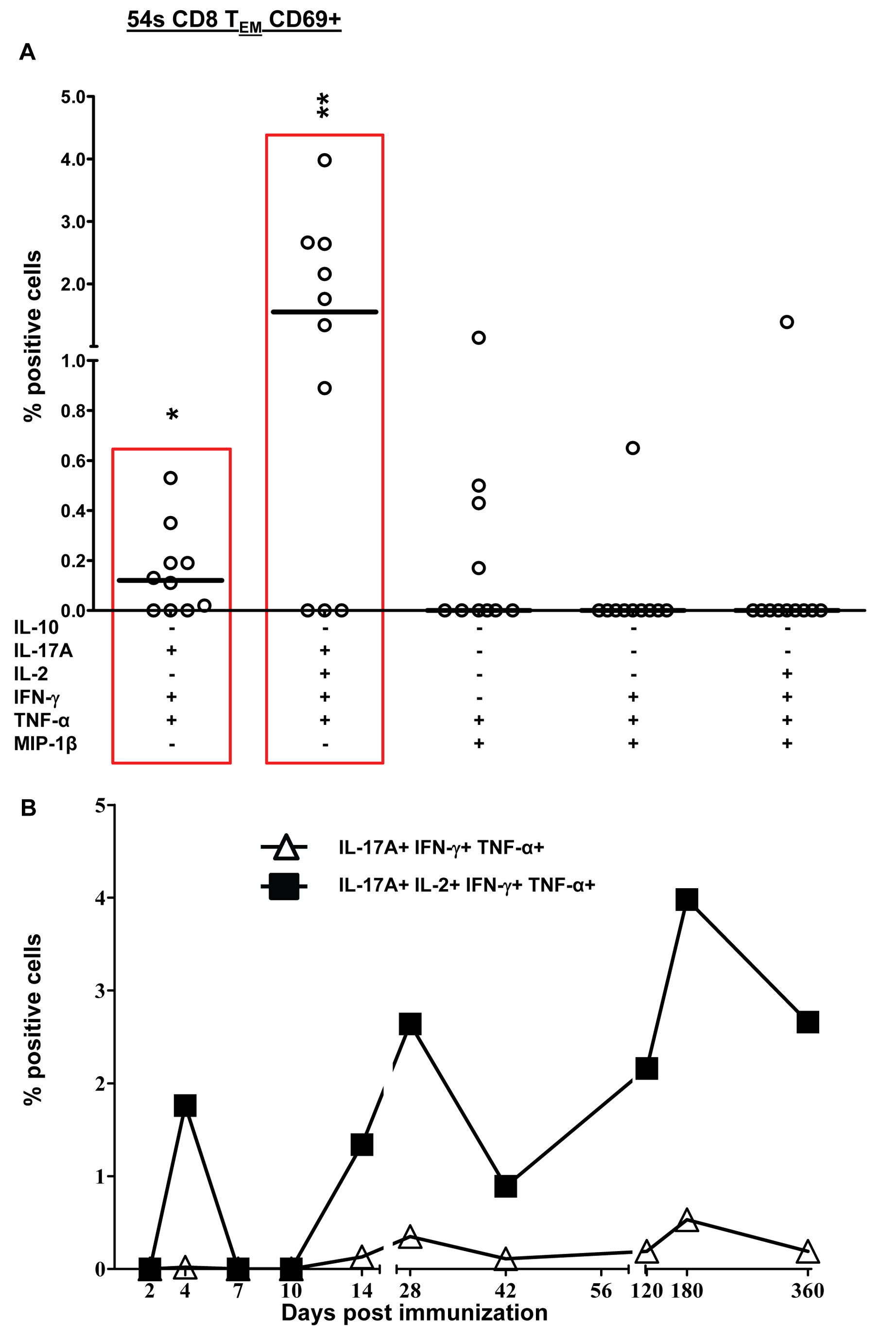

Supplement: Figure S4 — Multifunctional CD8+ TEM responses to S . Typhi-infected HLA-E restricted cells. A) Scatter plot showing all combinations of the 6 cytokines/chemokines measured that were positive at one or more time points for volunteer 54 s. Net increases of >0.5% cytokine positive cells over uninfected targets were found to be statistically significant (P<0.01). Each point represents a single time-point and the median value is denoted by a horizontal bar. The cyokine/chemokine combinations with the top 2 median values are indicated by red rectangles. Median values of the subsets of multifunctional cells that show a significant difference (*P<0.05, **P<0.01), when compared to the subsets without asterisks, are indicated. B) Kinetics of the top 2 populations (as determined by median value) over time. (TIF) [file pone.0038408.s004.tif]

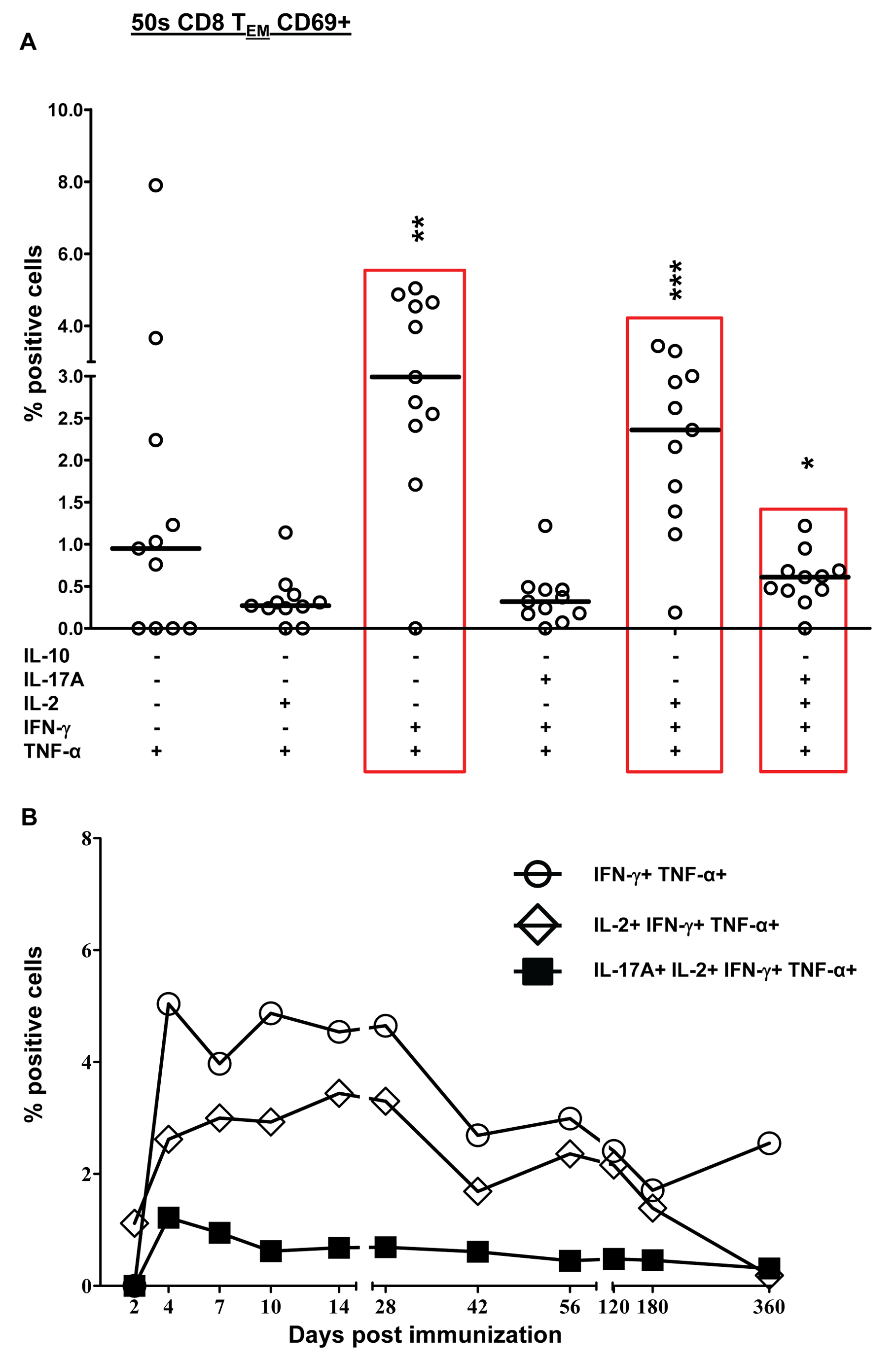

Supplement: Figure S5 — Multifunctional CD8+ TEM responses to S . Typhi-infected autologous B-LCL. A) Scatter plot showing all combinations of the 5 cytokines/chemokines measured (MIP-1β was not measured) that were positive at one or more time points for volunteer 50 s. Net increases of >0.5% cytokine positive cells over uninfected targets were found to be statistically significant (P<0.01). Each point represents a single time-point and the median value is denoted by a horizontal bar. The cyokine/chemokine combinations with the top 3 median values are indicated by red rectangles. Median values of the subsets of multifunctional cells that show a significant difference (*P<0.05, **P<0.01, ***P≤0.001), when compared to the subsets without asterisks, are indicated. B) Kinetics of the top 3 populations (as determined by median value) over time. (TIF) [file pone.0038408.s005.tif]
